# Metal Nanoparticle Film Deposition by Femtosecond Laser Ablation at Atmospheric Pressure

**DOI:** 10.3390/nano10112118

**Published:** 2020-10-25

**Authors:** Tony Donnelly, Gearoid O’Connell, James G. Lunney

**Affiliations:** 1 School of Physics and CRANN, Trinity College Dublin, The University of Dublin, Dublin 2, Ireland; oconnelg@tcd.ie; 2 School of Physics, University College Dublin, Belfield, Dublin 4, Ireland

**Keywords:** femtosecond laser ablation, plume expansion in gas, nanoparticle films

## Abstract

Nanoparticle gold films were deposited using femtosecond laser ablation in argon at atmospheric pressure in an arrangement where a flat Au target was irradiated through a transparent substrate in close proximity. Spatially extended films were made by rastering the target and substrate assembly together in the laser beam. Fast imaging clearly showed pronounced narrowing of the ablation plume, which can be understood in terms of laser induced multiphoton ionisation and heating of the gas near the ablation site. Deposition was possible for target-substrate separation up to 2 mm. The equivalent thickness of the nanoparticle film was controlled in the range 0.4–28 nm by changing the target-substrate separation and the shot-to-shot spacing of ablation spot raster. The mean Feret diameter varied in the range 14–40 nm depending on the deposition conditions, and all the films showed a surface plasmon resonance at about 525 nm, which was nearly independent of the equivalent thickness. The technique can readily be applied to other materials for the fabrication of nanoparticulate films at atmospheric pressure.

## 1. Introduction

There is continuing interest in the preparation and characterisation of nanostructured metal films with feature sizes in the range 1–100 nm. This interest is due to the many novel properties (optical, magnetic and catalytic) which arise when the dimensions of a solid material are reduced to the point where the particle contains tens to a few thousands of atoms [1]. Nanoparticle (NP) films of noble metals are of interest because they display a surface plasmon resonance (SPR) in the visible region, which can be used to enhance the sensitivity of fluorescence and Raman spectroscopies [2,3]. In the field of heterogeneous catalysis, spatially separated metallic nanoparticles on surfaces can exhibit pronounced size-dependent catalytic properties that differ considerably from those of single-crystal surfaces [4].

It has been shown that pulsed laser deposition (PLD) is a relatively simple and effective technique for deposition of metal NP films. Alfonso et al. (Ag and Cu on sapphire) [5,6], Dolbec et al. (Pt on highly oriented pyrolytic graphite) [4], Donnelly et al. (Ag and Au on glass, polymer and Si) [7,8] and Seal et al. (Ag on Si) [9] have all investigated PLD of metal NP films using nanosecond (ns) lasers in vacuum or low pressure background gases (<0.1 mbar). These studies have shown that nucleation and growth of metal NPs is dominated by processes occurring on the substrate rather than in the gas phase, and the NP size and shape depends on the deposited equivalent thickness and the energy of the ablated species respectively. Andrea et al. [3] and Smyth et al. [10] have investigated the deposition of Ag NPs using ns-PLD in a low pressure background gas and vacuum respectively, and demonstrated the utility of the films produced for surface enhanced Raman spectroscopy (SERS).

Femtosecond (fs) pulse duration lasers have also been used for PLD of NP films [11,12]. Liu et al. [13] have reported that fs-PLD of Ni in vacuum leads to polycrystalline NPs with an average diameter less than 10 nm, and this study and others have shown that the NP size is nearly independent of laser fluence [11,13]. Chakravarty et al. [14] have compared the formation of Ag and Cu NP films on Si substrates PLD in vacuum using picosecond (ps) and fs pulses. They observed that NPs with smaller mean diameter could be generated using fs-PLD compared to ps-PLD. In another experiment, De Bonis et al. [15] used fs-PLD to fabricate Ag NP films on solid substrate for SERS. They observed that the nanoparticle films produced by fs-PLD were very similar to the films produced by ns-PLD. Recently Mirza et al. [16] performed a comparison of ns and fs PLD of Ag and confirmed that NP growth is quite similar for the two laser pulse durations.

As for other thin film deposition techniques, PLD typically requires vacuum, or low pressure, conditions. In vacuum the free expansion of the plume of laser ablated material is well described by an isentropic adiabatic gas dynamical model [17]. When PLD is carried out in a low pressure background gas there is a complex interaction between the ablated material and the background gas that depends on the density of the background gas and the mass and kinetic energy of the ablation plume [18,19,20]. The plume is slowed, and eventually brought to a halt, by the background gas. For background gas pressure in the range of 0.01–1 mbar the plume typically propagates to distances ~ 3–8 cm. For higher gas pressures, the ablation plume is more confined to target, such that for typical ablation in gas at 1 bar, the maximum expansion of the plume is only 1–3 mm [21]. Thus, it is difficult to do PLD at atmospheric pressure, since moving the substrate close to the target tends to obstruct the laser beam. However, the possibility of doing PLD at atmospheric pressure is of interest, since avoiding the use of a vacuum chamber would facilitate the translation of PLD to industry for certain applications.

There are some reports of using laser ablation for deposition of materials at atmospheric pressure. Konov et al. [22] investigated atmospheric pulsed laser deposition (APLD) of diamond-like carbon by using a gas jet to assist the transport of the ablated material to the substrate. Nedyalkov et al. [23] studied APLD of Au NP films by reducing the target-substrate to 5 mm, and nano-porous films were synthesised. Khan et al. [24] showed that by using a jet of Ar gas directed across the ablation target it was possible to deposit silver NP films at up to 20 mm from the target in Ar at atmospheric pressure, and these films were effective for SERS. Cavaliere et al. [25] have recently carried out fs-PLD of TiO_2_ at ambient pressure and room temperature. In this study the substrate was placed at a 30° angle with respect to the target surface to allow access of the laser beam, and the target–substrate separation was 2–4 mm. In that study crystalline, fractal TiO_2_ nanostructures were deposited with high coverage and the nanostructure properties were shown to depend on the laser fluence and target substrate distance used. There are also several reports of APLD using various atmospheric plasma assist methods [26,27,28].

Laser induced forward transfer (LIFT) is another method of atmospheric deposition, where a donor coating on a transparent carrier plate is irradiated through the carrier plate with a pulsed laser so as to drive a small portion of the coating on to a receiver plate in close proximity. An example of this technique is described in reference [29], where arrays of sub-micron Cr structures were deposited. Zywietz et al. [30] have realised a similar technique for depositing Si nanoparticles on glass in ambient conditions, which does not require the preparation of a donor film as in LIFT. Rather, the substrate was placed at a distance of 13 μm from the Si target, and a fs laser irradiated the target through the substrate. The typical feature size was 0.1–1 μm, and it seems that the deposition is due to molten droplets expelled from the target. 

Previously we have reported on using a confined laser ablation geometry to make single-shot metal NP film deposition in vacuum [31]. A ns laser was used, and the NP deposit is comprised of 5–50 nm NPs. The confined ablation method has been used to make NP Au films at atmospheric pressure [32]. In that case a transparent polymer substrate was placed at 50 µm above a flat gold target, and a 10 kHz 700 ps laser was scanned over a 5 mm × 5 mm area. 

This paper describes the results of an experimental investigation of confined APLD in Ar using target-substrate separations up to 2 mm, and a fs laser to irradiate the target through the transparent substrate. Fast imaging and time- and space-resolved spectroscopy were used to reveal the expansion dynamics of the vapour and NP ablation plume, and to determine the plume stopping distance. Pronounced narrowing of the ablation plume was understood in terms of multiphoton ionisation by the laser beam, leading to the generation of a low-density channel in the gas, and lateral confinement of the ablation plume. The target and substrate were rastered together in the laser beam to obtain a uniform NP deposit. The solid-density equivalent thickness was controlled by changing the target-substrate separation and the step size of the raster system. Scanning electron microscopy was used to examine the film nanostructure. Optical absorption was used to determine the equivalent thickness and show the presence of the expected SPR. 

## 2. Materials and Methods

**Figure 1:**
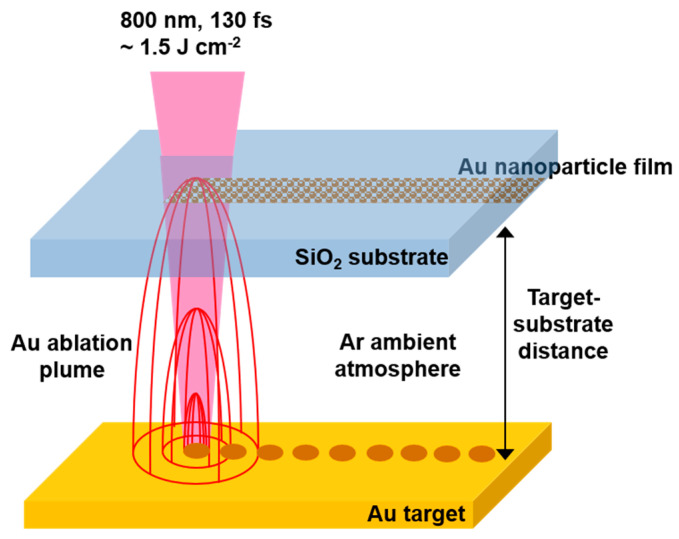
Target and substrate setup used for atmospheric pulsed laser deposition.

Figure 1 shows the experimental setup for APLD using a 130 fs, 800 nm Ti:sapphire laser (Coherent Legend Elite). A 10 mm × 10 mm, 1 mm thick quartz substrate (Pi-KEM) and a flat Au ablation target (GoodFellow) were mounted together with a 0.5, 1, or 2 mm thick spacer to form a simple deposition cell, with a channel for gas flow. The laser irradiation of the target was done through the substrate. The target and substrate were translated (ThorLabs Motorised X-Y Translation Stage) together in the plane orthogonal to the laser beam to produce deposition over a 5 mm × 5 mm area. The region between the target and the substrate was filled with Ar at atmospheric pressure. Laser pulses of energy 0.5 mJ were focused to a Gaussian spot using a 30 cm lens at normal incidence. The focused spot was slightly elliptical with 1/e^2^ diameters of 360 and 330 µm, yielding a maximum fluence of ~1.5 J cm^−2^ (irradiance ~1 × 10^13^ W cm^−2^). The laser fluence was below the breakdown threshold of the SiO_2_ substrate. Depth analysis of the ablation crater (Zygo Optical Profilometer) formed by 50 laser shots at the same position revealed that the maximum ablation depth per shot was ≈100 nm, and the ablated mass was 23 ng, which corresponds to 7 × 10^13^ atoms ablated per pulse. The major and minor ablation crater diameters were 220 and 180 µm. For most of the depositions the target and substrate were rastered together in the laser beam to yield shot-to-shot separation of 250 µm in both directions. In this case there is no overlap of the ablation craters, and each deposition event is spatially isolated from neighbouring events. For a shot-to-shot spacing of 250 µm, the laser repetition rate was 1 Hz, and translation speed was 0.25 mm s^−1^. After each line scan the stage moved laterally so that the shot-to-shot spacing was the same in both directions (i.e., 250 µm in this case). 

To investigate the effects of partial overlap of the ablation craters and regions of deposition the repetition rate was increased to 3 and 10 Hz while keeping the translation speed at 0.25 mm s^−1^. This produced a shot-to-shot spacing of 80 and 25 µm, with pulse overlaps of ~60% and ~80%, respectively. At these conditions each site was irradiated approximately three and nine times respectively, and the corresponding depositions were overlapped. The motivation here was to increase the deposition thickness above the level where the laser shots were spatially separated, but it was expected that there would be some interaction of the laser pulses with material deposited by previous shots. The dynamics of the ablation plume expansion without the substrate was investigated in vacuum, and at various Ar pressures up to 1 bar, using time-resolved imaging of the ablation plume optical self-emission with an imaging system and intensified charged couple detector (iCCD) (Andor iStar) viewing the plume in a direction parallel to the target surface. Time- and space-resolved emission spectra of the ablation plume were recorded using a 0.25 m Czerny-Turner spectrometer (Newport Oriel MS260i) equipped with a 300 lines per mm grating and an iCCD (Andor iStar). A 0.5× demagnified image of the plume was formed on the 50 µm wide spectrometer slit, with the z-axis of the plume image lying along the slit. Thus, the recorded spectrum was due to a 100 µm wide region of the plume centred on the z-axis. The spatial resolution of the spectrum in the direction normal to the target surface was ~70 µm.

The NP deposits were examined using a scanning electron microscope (SEM) (Zeiss Ultra Plus), though the resolution was limited by charging of the insulating substrates. A low accelerating voltage between 0.5–1.2 kV was used to minimise surface charging and drift during image capture. Both secondary and backscattered electron detectors were used for imaging, with the latter affording better elemental contrast at the expense of spatial resolution. Size distributions were extracted from the captured images using statistical counting in the ImageJ software package (Version 1.53a) [33]. The spectral absorbance of the NP Au deposits was measured in the range 200–800 nm, using a bare quartz substrate for reference. The equivalent solid density thickness was estimated from the measured optical absorbance at 300 nm, where according to Haiss et al. [34] the optical absorbance is mainly due to bound electrons and not much influenced by the nanostructure.

## 3. Results and Discussion

**Figure 2:**
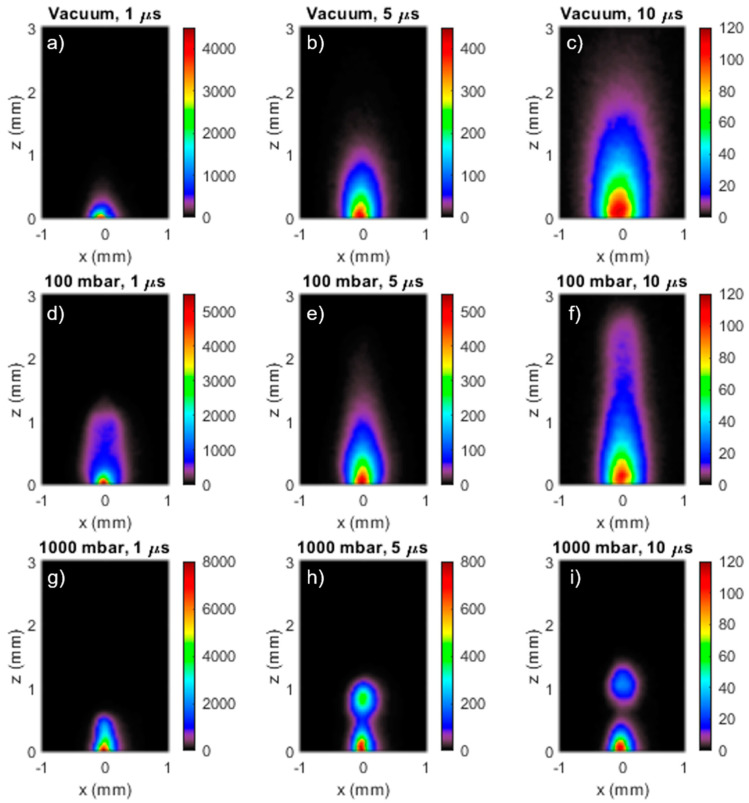
Images of the ablation plume optical emission in vacuum (**a**–**c**) at 1 µs, 5 µs and 10 µs after the laser pulse, respectively, in 100 mbar Ar (**d**–**f**) at 1 µs, 5 µ and 10 µs, respectively, and in 1 bar of Ar (**g**–**i**) at 1 µs, 5 µs and 10 µs, respectively. The scale bar units are counts per ns.

In the first instance the gas pressure dependence of the ablation plume expansion was examined by recording iCCD images of the ablation plume at variable delays after the laser pulse for ablation in vacuum, 100 mbar, and 1 bar of Ar, as shown in Figure 2. In these images the x-axis is parallel to the target surface, and the z-axis is normal to the target surface. The laser fluence was 1.5 J cm^−2^, and the iCCD gate time was set to be 5% of the delay between laser irradiation and iCCD acquisition. The images in Figure 2a–c was recorded for ablation in vacuum at 1 µs, 5 µs and 10 µs after the laser pulse. The plume component furthest from the target consists of plasma formed by direct vaporisation of a thin layer of material near the target surface [35], and at 1 µs (Figure 2a) it has expanded beyond the 3 mm extent of the imaged region in front of the target. The second brighter component lying closer to the target surface is a plume of hot, liquid phase NPs of the target material. This component originates from a hot dense layer at 5–15 nm below the target surface which unloads from the target as nano-particulate matter [35]. The images in Figure 2b,c shows that the NP plume is expanding at about 100 m s^−1^.

Much theoretical and experimental work has been done to understand the origin and physical properties of the two plume components and has been reviewed in reference [36]. Essentially, the formation of the NP plume is due to a combination of fragmentation and phase explosion mechanisms. Fragmentation is mechanical breakup due to the high strain rates associated with rapid material expansion. Phase explosion occurs when expansion carries the laser heated material under the bimodal line in the phase diagram, where homogenous bubble nucleation occurs, leading to NP formation. Experimental confirmation of the presence of the different plume constituents has been obtained using optical emission spectroscopy [35] and x-ray absorption spectroscopy [37]. The velocity of the plasma component has been previously measured to be ~10^4^ m s^−1^ in vacuum, whilst the NP plume velocity was measured to be approximately 100× slower at ~100 m s^−1^ [35].

Figure 2d–i shows the influence of increasing gas pressure on the expansion of the plasma plume. For 100 mbar the confinement of the plasma plume was already evident at 1 µs (Figure 2d), where it had a semi-ellipsoidal shape extending to about 1.2 mm in front of the target. The images at 5 µs (Figure 2e) and 10 µs (Figure 2f) show the plasma plume continuing to expand in the direction normal to the target, reaching nearly 3 mm at 10 µs. However, it is also very clear that lateral expansion of the plume was less than expected, such that at 10 µs the plume had a nearly cylindrical shape with a radius of only about 0.3 mm. For fs laser ablation in gas, this plume narrowing can be understood in terms of laser induced multiphoton ionisation, leading to heating the gas in front of the target [38]. The radial expansion of this heated gas leads to the formation of a low-density plasma pipe into which the ablation products are channelled, giving rise to plume narrowing. Thus, the ablation plume for fs ablation in gas could be narrower and longer compared to ns ablation, where the laser irradiance was too low to cause multiphoton ionisation. Figure 2g–i shows plume images for ablation in 1 bar of argon at 1 µs, 5 µs, and 10 µs. Compared to 100 mbar, both the plasma and nanoparticle plumes were more strongly confined near to the target surface. The image at 5 µs shows that the expansion of the plasma plume into the low-density channel led to the formation of a globular region of enhanced emission at about 0.8 mm from the target. This globular region moved out to 1.1 mm at 10 µs. Most likely, formation of this globular emission was due to the accumulation of the plasma plume at the end of the plasma pipe. The extension of the plasma pipe in front of the target was determined by how the radius of the laser beam increased away from the focus, leading to a decrease of the laser irradiance, and thus the degree of multiphoton ionisation [38]. Images recorded at 50 µs (not shown here) showed that the maximum extent of the NP plume was about 0.5 mm.

**Figure 3:**
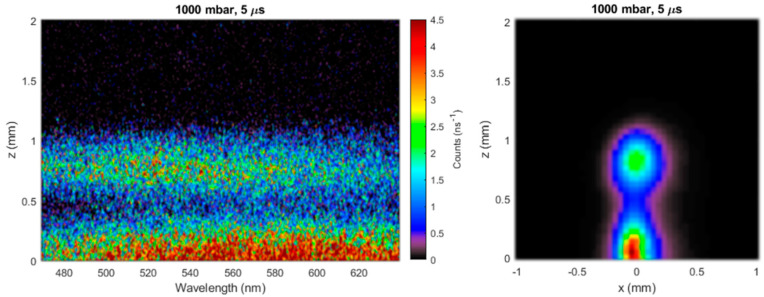
Space-emission spectra (**left**) of the two ablation plume features, together with the corresponding plume images (**right**), acquired at 5 µs after the laser pulse.

Some further insight into the dynamics of plume expansion in fs ablation in gas was revealed by space-resolved visible emission spectroscopy. Figure 3 shows a space-resolved emission spectrum, together with the corresponding image, taken at 5 µs. The NP plume displayed the expected continuum emission due to the grey body emission of the hot nanoparticles with a temperature of about 2400 K. The globular region, which was derived from the confined plasma plume, also showed continuum emission. It seems likely that the confinement of the plasma plume led to NP condensation. Then, according to this interpretation, the shape of the grey body spectrum corresponded to a NP temperature of about 2800 K.

**Figure 4:**
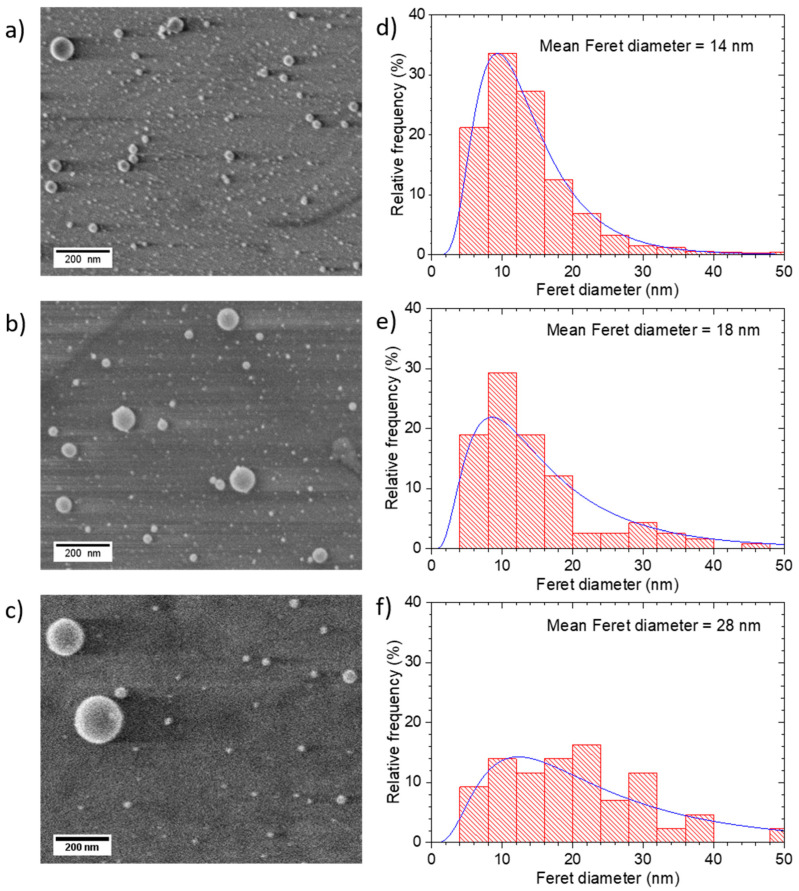
SEM images of 1 × 1.2 μm^2^ area of the Au nanoparticle films deposited at substrate distances of (**a**) 0.5, (**b**) 1 and (**c**) 2 mm, together with the corresponding Feret diameter distributions, (**d**–**f**) respectively.

Figure 4a–c shows SEM images of Au NP films deposited at 1 bar of argon with the SiO_2_ substrates posited at 0.5, 1 and 2 mm from the ablation target, respectively. The target–substrate combination was translated 250 μm between laser shots to obtain single-shot deposition, as described in the Methods and Materials section. The SEM images were recorded at the centres of deposition sites. All three target–substrate separations showed well separated NPs. Clearly the deposition became sparser as the target–substrate separation was increased from 0.5 mm to 2 mm. This observation is consistent with the plume images taken at atmospheric pressure (Figure 2g–i). The NP size distributions also depended on target-substrate distance, as can be seen in the SEM images, and in the Feret diameter distributions shown in Figure 4d–f. The Feret diameter increased from 14 nm at 0.5 mm, to 18 nm at 1 mm, and 28 nm at 2 mm.

**Figure 5:**
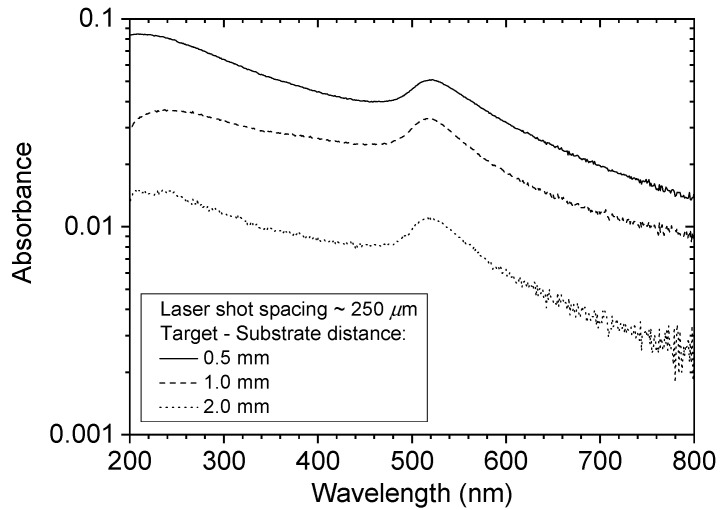
UV/visible absorbance spectra of films deposited at 0.5 mm, 1.0 mm and 2.0 mm separation, using a laser shot spacing of 250 µm.

The decrease in the amount of deposition with increasing target-substrate separation was also revealed by measuring the spectral absorbance of the films. Figure 5 shows UV/visible absorbance spectra of films deposited at 0.5 mm, 1.0 mm and 2.0 mm separation, using a laser shot spacing of 250 µm, which corresponds to single-shot deposition. The spectra showed the expected peak in absorbance at about 520 nm due to the SPR of the free electrons in the NPs. The amplitude of the plasmon peak decreased as the target-substrate separation was increased from 0.5 mm to 2 mm, consistent with a reduction of the fraction of the ablated material deposited. Haiss et al. [34] investigated the optical absorption properties of gold NPs of various sizes in liquid suspension in the spectral region 300–900 nm. As expected, it was observed that the wavelength of the SPR absorbance peak depended on the NP size. The SPR peak at 520 nm for the NP sizes shown in Figure 4 is consistent with the results in reference [34]. In that paper it is also noted that the absorption at 300 nm is mainly due to bound, rather than free, electrons, and is not much influenced by the nanostructure. Thus, the optical absorbance at 300 nm can be used to estimate the solid-density equivalent thickness of the Au NP films, and corresponding absorbance per nm is 0.031 nm^−1^. From the absorbance values in Figure 5 the solid-density equivalent thickness values were determined to be 2.1 nm, 1.1 nm and 0.37 nm for the films made at 0.5 mm, 1.0 mm and 2.0 mm, respectively. It was noted above that the mass ablated per pulse was 23 ng. If this amount of Au was spread evenly over a 250 µm × 250 µm area, corresponding to the raster step size, the equivalent thickness was 1.65 nm. This value was of the same order as the measured value of 2.1 nm for the films made at a 0.5 mm separation, indicating that most of the ablated material was deposited at this position.

The maximum distance for the transmission of the NP plume through argon gas at 1 bar can be estimated in terms of the physical description of the slowing down of a spherical particle in gas. The slowing down of fs laser-generated platinum NPs has been studied for argon pressures up to 51.7 Torr [39]. Consider the slowing down of a spherical particle of radius *r* moving in a gas where the mean free path of the gas molecules is *λ.* When r≪λ the particle is slowed down by the collective effects of collisions with individual molecules, and the slowing down is described by the Epstein drag force, which depends linearly on the relative velocity between the particle and the gas. Following the nomenclature in Ref. [39], the position *x*(*t*) of a particle launched at velocity *v*_0_ is given by $x\left(t\right)=x_{\infty }\left(1-e^{-\alpha t}\right),$ where *x*_∞_ is the stopping distance and *α* is the slowing down parameter. This is given by $\alpha =\frac{4\delta P}{D\rho }\sqrt{\frac{2M}{\pi RT},}$ where the value of *δ* is close to 1 + π/8 for metal particles, *P* is the gas pressure, *D* and *ρ* are the diameter and density of the spherical particle, respectively, *M* is the molar mass of the gas, *R* is the gas constant, and *T* is the gas temperature. The stopping distance *v*_∞_ is given by $v_{\infty }=\frac{v_{0}}{\alpha }$. For gold NP with a typical diameter of 30 nm the stopping parameter is *α* = 2.8 × 10^7^ s^−1^, and for $v_{0}$ = 100 m s^−1^ the stopping distance is only 33 µm. However, the images in Figure 4 show that NPs could be deposited on a substrate at up to 2 mm from the target. It seems that this apparent contradiction was due to the laser induced plasma tube having a much lower density of gas molecules than the ambient gas. Thus, it seems that the production of the plasma tube facilitated NP disposition at a much greater distance than would otherwise be the case. It can also be noted that since the stopping distance was linearly dependent on the particle diameter, it was expected larger particles would propagate further than smaller particles. This tendency is apparent in the images in Figure 4 where the film made at 0.5 mm had a higher preponderance of small NPs as compared to the film made at 2 mm.

**Figure 6:**
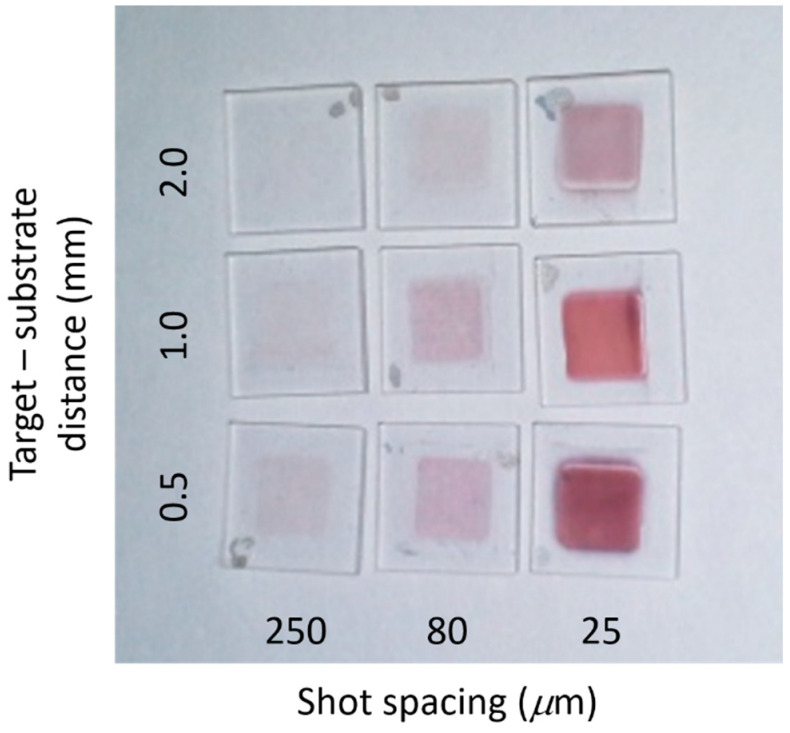
Photograph of the nanoparticle (NP) films deposited at different values of target-substrate separation and laser shot spacing.

It is of interest to explore the extent to which the equivalent thickness of the NP films can be increased by reducing the shot-to-shot spacing below 250 µm, which is the minimum value for having little interaction of the laser beam with the deposit made by the previous pulse. To explore this aspect the laser repetition rate and translation stage speed were adjusted to give shot-to-shot spacing of 25 μm (strong overlap), and 80 μm (moderate overlap). Figure 6 shows a transmitted light photograph of the depositions made for shot spacings of 250 µm, 80 µm, and 25 µm with target-substrate separations of 0.5 mm, 1.0 mm and 2.0 mm. All the depositions showed the usual red colour indicative of nanoparticulate gold. Clearly the equivalent thickness increased with reducing the shot spacing and the target-substrate separation.

**Figure 7:**
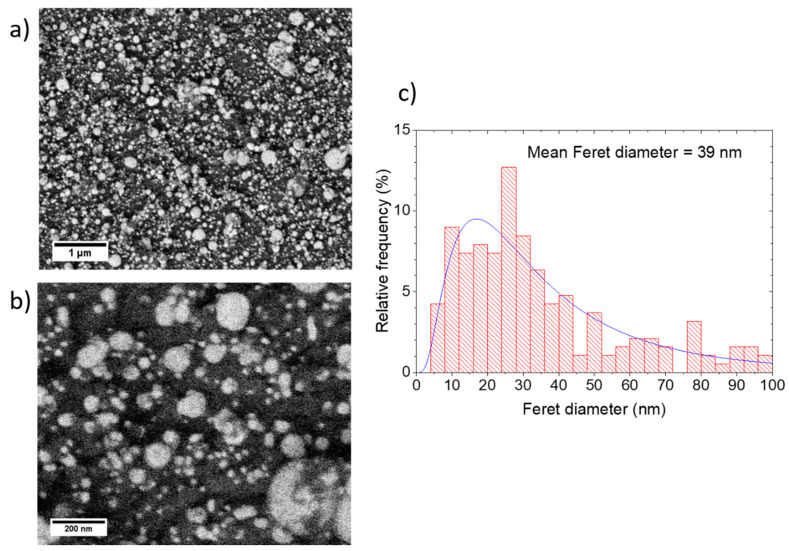
SEM images of a film made at 1 mm target-substrate separation, 25 µm laser spot spacing (**a**,**b**), and the corresponding Feret diameter distribution (**c**).

**Figure 8:**
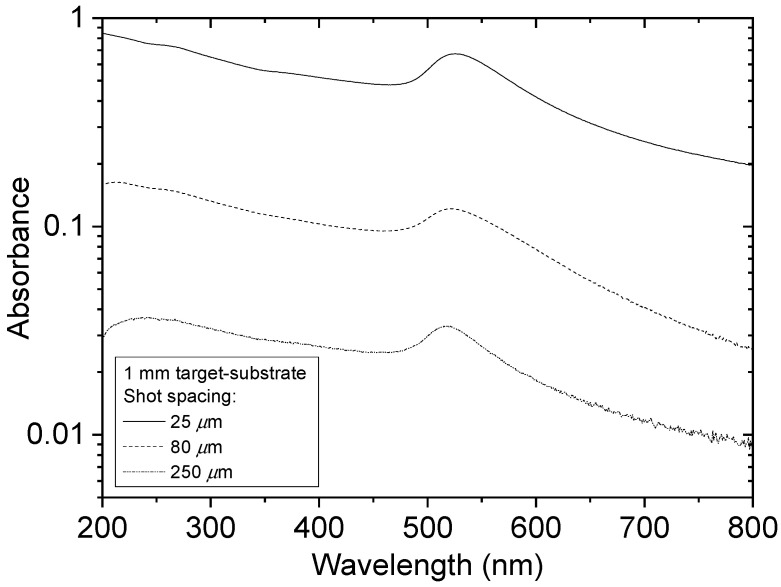
Optical absorbance spectra of Au NP films deposited at a target-substrate separation of 1 mm and with laser spot spacing of 25 µm, 80 µm, and 250 µm in each direction.

Figure 7 shows SEM images and the Feret diameter distribution for the film made at 25 µm shot spacing and 1 mm target-substrate separation. The images show a densely packed NP film, and the mean Feret diameter was 39 nm. Figure 8 shows how the optical absorbance increased as the shot spacing was decreased from 250 µm to 25 µm for deposition with the substrate at 1 mm from the target. The SPR peak was clearly observed in each case and did not shift to longer wavelength as the equivalent thickness increased. This contrasts with vacuum PLD of NP films where a shift to longer wavelength with increasing thickness was observed [16].

**Figure 9:**
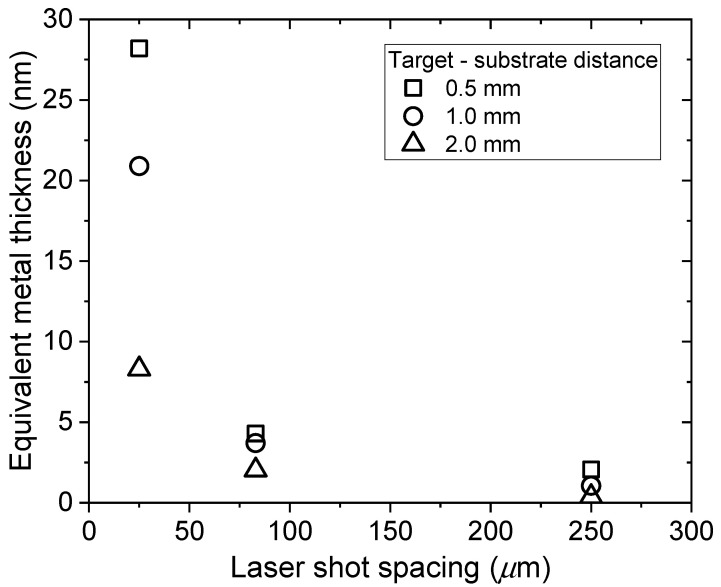
Equivalent thickness of Au NP films deposited at atmospheric pressure using different values of target-substrate separation and laser shot spacing.

As described above, the optical absorbance at 300 nm was used to determine the equivalent thickness of films made at the different values of shot spacing and target-substrate separation, and the results are shown in Figure 9. For 25 µm shot spacing and 0.5 mm target-substrate separation, NP films with equivalent thickness of 28 nm were obtained, and even at this thickness the SPR was observed. Thus, this atmospheric pressure process can be adjusted to produce, in a single pass, NP films across a thickness range of 0.1–30 nm. However, laser absorption in already deposited material will determine an upper limit on the achievable equivalent thickness. For example, the film made at 25 µm spot spacing and 1 mm target-substrate separation is 20.9 nm thick and the measured transmission at 800 nm is 64%. Depending on the fluence in the incident beam, above a certain deposited thickness absorption will reduce the fluence at the target to below the ablation threshold. When there is significant overlap of the laser ablation spots, material already deposited will be subject to laser irradiation at a level sufficient to cause ablation of the solid gold target. It is expected that deposited material in the path of the laser beam will be vapourised and subsequently condensed. Thus, for ablation with significant laser spot overlap, the deposited material will have undergone several cycles of condensation and evaporation before eventually moving out of the laser beam path.

## 4. Conclusions

A new fs laser ablation technique for the deposition of gold NP films in argon at atmospheric pressure was investigated. To overcome the problem of plume confinement in gas, the laser was incident normally on a flat gold target through a transparent substrate in close proximity. The target and substrate assembly were rastered together in the laser beam to deposit a spatially extended coating. Fast imaging clearly showed pronounced narrowing of the ablation plume, which can be understood in terms of laser induced multiphoton ionisation and heating of the gas, leading to the formation of a low density channel in the gas into which the ablation plume is channelled. The effect leads to the propagation of nanoparticulate material through much thicker regions of gas than would be expected from the Epstein viscous drag. It was demonstrated that the equivalent thickness of the NP can be controlled in the range 0.4–28 nm by changing the target-substrate separation and the shot-to-shot spacing of ablation spot raster. The mean Feret diameter varied in the range 14–40 nm depending on the deposition conditions. All the films displayed a SPR at wavelength of about 525 nm, which was nearly independent of the equivalent thickness. The technique described here can readily be applied to other optically absorbing materials, such as other metals and semiconductors, for the disposition of NP films on transparent substrates without needing to use a vacuum chamber or wet chemistry.
